# Incidences of underlying causes of hypothermia in older patients in the emergency department: a systematic review

**DOI:** 10.1007/s41999-023-00791-0

**Published:** 2023-05-16

**Authors:** Birgitta M. G. Snijders, Marvin J. Roos, Carolina J. P. W. Keijsers

**Affiliations:** 1 Department of Geriatrics, University Medical Center Utrecht, Heidelberglaan 100, 3584 CX Utrecht, The Netherlands; 2 Department of Geriatrics, Jeroen Bosch Hospital, Henri Dunantstraat 1, 5223 GZ ‘S-Hertogenbosch, The Netherlands

**Keywords:** Hypothermia, Etiology, Emergency department, Older patients, Systematic review

## Abstract

**Purpose:**

Hypothermia is a serious condition in older adults. Knowledge of a priori chances of underlying diseases may affect initial management, hence prognosis. This systematic review provided an overview of existing literature on the incidences of underlying causes of hypothermia in older patients at the emergency department.

**Methods:**

MEDLINE, The Cochrane Library, and Embase were searched up to February 1st, 2022. Inclusion criteria were age ≥ 65 years, emergency department setting, and body temperature < 36.0 degrees Celsius. Exclusion criteria were iatrogenic hypothermia, no underlying cause reported, and patient selection based on specific diseases. Title/abstract and full-text were screened and quality was assessed using the Joanna Briggs Institute Critical Appraisal Tool. Data were presented using descriptive statistics and narrative analyses.

**Results:**

Forty-one reports were included, including 6 cohort studies and 35 case reports. The 6 studies involved 2173 hypothermic patients, whose age varied from a mean of 67 to a median of 79 years and temperature from a median of 30.8 to a mean of 33.7 degrees Celsius. One study reported about primary hypothermia (incidence of 44%). Acute medical illness was often reported as underlying cause of secondary hypothermia (49–51%). Reported incidences of infection and sepsis ranged from 10 to 32%, of trauma up to 14%, and of alcohol intoxication from 5 to 26%.

**Conclusion:**

Limited studies have been published regarding this topic, and the overall quality of the evidence was graded as low. Causes that should not be missed include acute medical illness, trauma, alcohol intoxication, primary hypothermia, thyroid failure, and drug-induced hypothermia.

**Supplementary Information:**

The online version contains supplementary material available at 10.1007/s41999-023-00791-0.

## Key summary points

**Aim:**

This systematic review provides an overview of existing literature on the incidences of underlying causes of hypothermia in older patients at the emergency department.

**Findings:**

Underlying causes of hypothermia that should not be missed include infectious, endocrine, neurological, cardiac and gastro-intestinal diseases, trauma, alcohol intoxication, primary hypothermia, and drug-induced hypothermia.

**Message:**

Knowledge of the a priori chances of underlying causes of hypothermia in older patients presenting to the emergency department may affect initial management, hence prognosis.

**Supplementary Information:**

The online version contains supplementary material available at 10.1007/s41999-023-00791-0.

## Introduction

Research showed that older patients are more vulnerable to develop hypothermia [1]. Hypothermia is associated with a high risk of morbidity and mortality, and these risks rapidly increase with age [2]. For example, a recent study showed that 1-year mortality of all-cause hypothermia significantly increased with age from 1.7% in patients aged 18–20 years up to 45% in patients aged > 80 years [3]. Furthermore, frailty is associated with higher mortality rates [4]. This makes hypothermia a serious problem in older patients. Prompt recognition and early treatment of underlying causes of hypothermia are needed in order to minimize negative health outcomes. For example, it is known that a rapid administration of antibiotics lowers the risks of adverse outcomes in septic patients [5]. However, hypothermia is often associated with diagnostic delay in septic patients [6]. Therefore, it is important to know the a priori chances of underlying diseases in hypothermic older patients presenting to the ED, as this may affect initial management, hence prognosis.

However, only a small number of studies have been published reporting the incidences of underlying causes of hypothermia. A Danish study reported accidental hypothermia to be the most prevalent cause of hypothermia and alcohol intoxication to be the most recorded secondary diagnosis [3]. In contrast, a Japanese study showed acute medical illness to be most frequently associated with hypothermia [1]. As most studies were performed in only one country, which has its own climate and living circumstances, the underlying incidences of hypothermia may vary per region, thus resulting in a low applicability to older ED patient in general. In order to assist physicians worldwide in clinical decision making, an overview of the existing literature is needed.

To our knowledge, no systematic review has yet been published regarding the incidences of hypothermia in older patients presenting to the ED. This study performed a systematic search aiming to identify how the underlying causes of hypothermia are distributed in older patients at the emergency department.

## Methods

### Search strategy

This systematic review was conducted and reported in accordance with the Preferred Reporting Items for Systematic Reviews and Meta-Analyses (PRISMA) guidelines using the ‘PRISMA 2020 Statement’ [7]. The completed PRISMA checklists were included in Appendix 1. The study protocol was registered in PROSPERO international prospective register of systematic reviews (registration number CRD42021246579). Patients or the public were not involved in the design, or conduct, or reporting, or dissemination plans of our research.

A systematic search of the electronic bibliographic databases MEDLINE, The Cochrane Library, and Embase was carried out. The search strategy included the terms and synonyms of ‘Old’ AND ‘Hypothermia’ AND ‘Emergency Department’. The complete search strategy per database can be found in Appendix 2. The geriatric search filter created by De Glind et al. was used as part of the search strategy [8].

### Study selection

Study selection was performed using the pre-established inclusion and exclusion criteria. Studies were included if: (1a) all participants were aged 65 years or above, (1b) at least part of the study population was aged ≥ 65 years and sufficient data were provided in order to extract results solely regarding patients ≥ 65 years, or (1c) insufficient data were provided to extract results regarding older patients, but the median or mean age of the whole study population was ≥ 65 years. Other inclusion criteria were: (2) it concerned ED patients with (3) hypothermia. Hypothermia was defined as a body temperature measurement < 36.0 degrees Celsius. If only part of the study population was hypothermic, sufficient data had to be provided in order to extract results solely regarding the hypothermic patients. Although hypothermia is often defined in the literature as a body temperature below 35 degrees Celsius, this review used a cutoff of 36 degrees Celsius in order to minimize the risk of missing relevant articles who may have used different body temperature thresholds [9]. Exclusion criteria were: (1) articles written in other languages than English, Dutch, or German, (2) medically induced hypothermia, (3) articles that did not report anything about the underlying causes of hypothermia, (4) studies whose patient selection was based on the presence of a specific disease, for example when only submersion injuries were included (except for case reports), and (5) cardiac arrest at ED arrival.

Screening of title, abstract and full text was consequently done independently by two reviewers (MR and BS). Disagreement was resolved through discussion between the two reviewers. If necessary, a third reviewer was consulted (CK). Reference lists of eligible studies were searched for additional references. Unpublished studies were not actively sought. No restrictions regarding study design or publication period were applied. The search was rerun prior to final analysis on February 1st, 2022.

### Quality assessment

Quality of the included studies was assessed by evaluating risk of bias. This was done by two researchers independently (MR and BS). Disagreement was resolved through discussion between the two reviewers. If necessary, a third reviewer was consulted (CK). Risk of bias was assessed for cohort studies using the validated Joanna Briggs Institute (JBI) Critical Appraisal Tool for Cohort Studies [10]. Studies were considered of poor quality if ≥ 2 questions were answered with ‘No’, if ≥ 1 question was answered with ‘No’ and ≥ 2 with ‘Unclear’ or if ≥ 3 questions were answered with ‘Unclear’. Studies were considered of medium quality if 1 question was answered with ‘No’ or if 2 questions were answered with ‘Unclear’. Studies were considered of high quality if all questions were answered with ‘Yes’ or if a maximum of 1 question was answered with ‘Unclear’. Quality of individual case reports was not assessed. Due to the low amount of available evidence, no studies were excluded from further analysis based on level of quality. Overall quality of the evidence was assessed using the Grading of Recommendations, Assessment, Development and Evaluations (GRADE) framework [11].

### Data collection and statistical analysis

Data were extracted from the included studies using a pre-established Excel sheet. Extracted data included study design, study population and sample size, age, country of study site, season, body temperature (if necessary converted to degrees Celsius), method of temperature measurement, and underlying causes of hypothermia with its incidence. Data were extracted by two researchers independently (MR and BS). Disagreement was resolved through discussion between the two researchers.

Data were presented using descriptive statistics and narrative analyses. Heterogeneity between studies was assessed to evaluate whether it was appropriate to perform a meta-analysis. A meta-analysis would be performed in order to estimate pooled disease frequencies in hypothermic older patients.

## Results

**Fig. 1:**
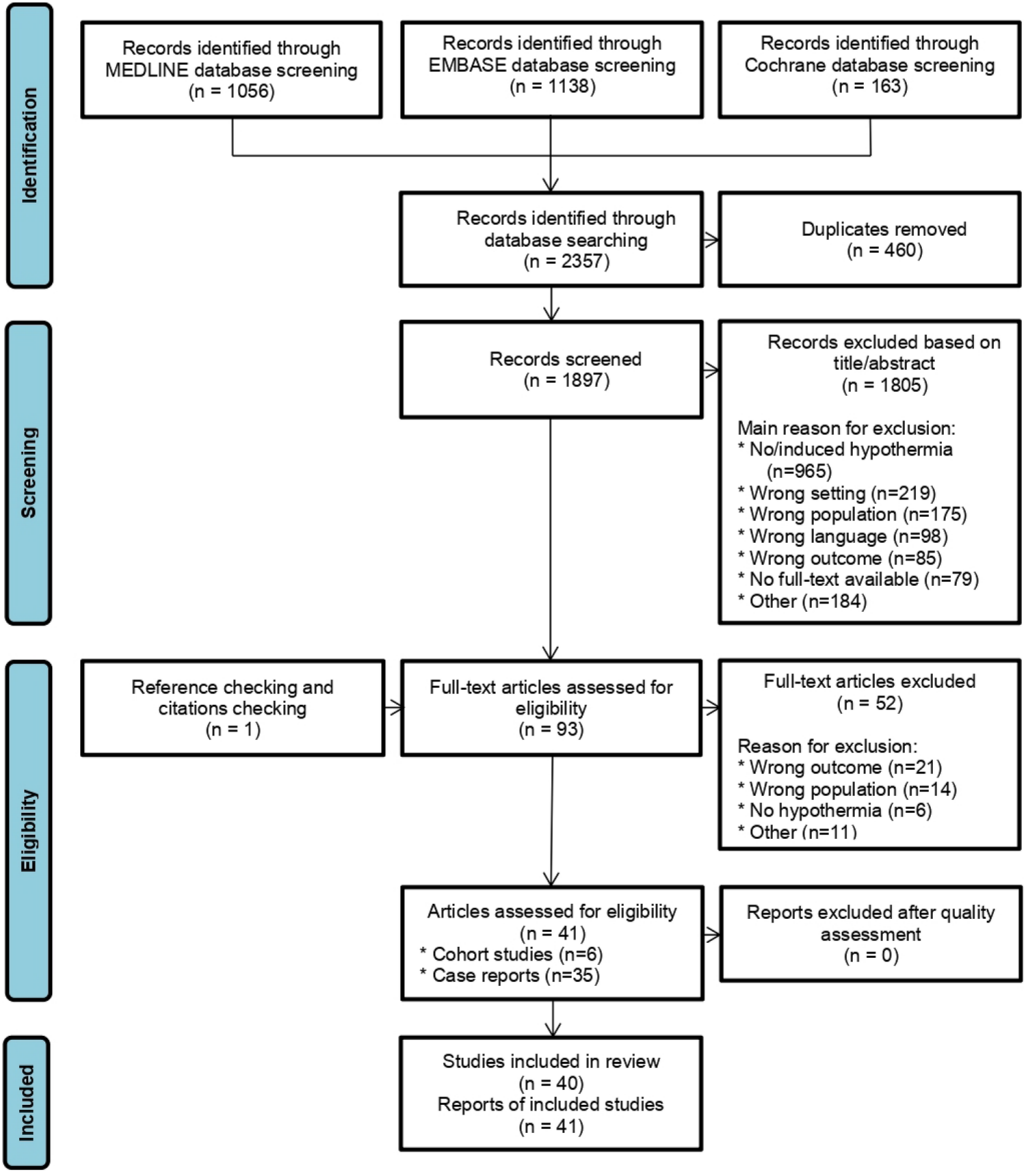
Flow chart of study selection

**Fig. 2:**
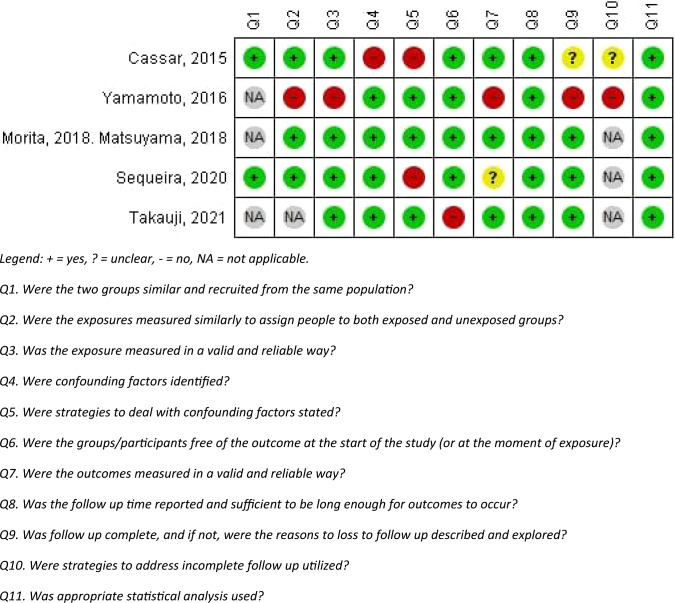
Risk of bias assessment

The literature search yielded 1897 unique articles, of which 1805 were excluded based on title and abstract and an additional 52 after full-text screening (Fig. 1). A total of 41 reports were included in this systematic review, which included 6 articles regarding 5 patient cohorts and 35 case reports and conference abstracts. The cohort studies were assessed for quality and only the studies of Morita et al. [2] and Matsuyama et al. [1] were considered high quality. The studies of Sequeira et al. [12] and Takauji et al. [13] were determined to be of medium quality and the studies of Cassar et al. [14] and Yamamoto et al. [15] to be of poor quality (Fig. 2).

### Cohort studies

The six cohort studies involved 3046 ED patients, of which 2173 were hypothermic (Table 1). All but one study had a retrospective study design and these data were collected using patient medical records [1, 2, 12, 14, 15]. The prospective cohort study of Takauji et al. did not clearly describe how the underlying cause of hypothermia was determined [13]. Three out of five study populations were recruited in Japan. The age of the included patients varied from a mean of 67 to a median of 79 years. Hypothermia ranged from a median of 30.8 to a mean of 33.7 degrees Celsius. There was a wide variety between studies on how the underlying causes of hypothermia were reported. Solely the study of Cassar et al. [14] reported data regarding the incidences of primary hypothermia, which was present in 44% of patients. Underlying causes of secondary hypothermia were classified differently in each study. Acute medical illness was often reported as underlying cause of secondary hypothermia (49–51%), which included infectious, endocrine, neurological, cardiac and gastro-intestinal diseases. Of studies that reported on infection and sepsis as the underlying cause of hypothermia, the incidences ranged from 10 to 32%. Trauma as underlying cause was reported in up to 14% of patients. Alcohol intoxication varied from 5 to 26%, although the incidences appeared to be considerably lower in the Japanese studies of Morita et al. [2], Matsuyama et al. [1], and Takauji et al. [13] (9% and 5%, respectively) compared to the American study of Sequeira et al. [12] (26%).

**Table 1:** Cohort studies of etiology of hypothermia in older patients at the emergency department

First author, year	Study design	Country (season)	Number of participants	Study population	Age in years (median (IQR))	Definition of hypothermia	Body temperature at ED arrival (°C)	Underlying cause of hypothermia
Cassar, 2015 [14]	Retrospective cohort	Malta (winter)	199	ED patients aged > 16 years who were hospitalized due to hypothermia	Not reported 96% (191/199) of patients were aged 60 years or older	< 35 °C measured rectally	32–35 °C: 40–55% 28–32 °C: 31–53% < 28 °C: 4–7%	Whole cohort,* n *= 199: Primary hypothermia: 44% Sepsis: 32% Decreased mobility: 21% Hypothyroidism: 3%
Yamamoto, 2016 [15]	Retrospective cohort	Japan (whole year)	913 Sub-group of hypothermic patients: n = 40	ED patients aged > 15 years who were hospitalized, had blood cultures drawn, and were ultimately diagnosed with bacterial infection	Whole cohort: 77 (65–83), Hypo-thermic patients: 78 (69 – 86)	< 36 °C measured at the axilla. If < 35 °C, measured using a bladder or rectal probe	Not reported	All hypothermic patients,* n*= 40: Pneumonia: *n* = 21 (53%) UTI: *n* = 5 (13%) SSTI: *n* = 5 (13%) Acute cholangitis: *n* = 1 (3%) Acute cholecystitis: *n* = 1 (3%) Other bacterial infections: *n* = 7 (18%)
Morita, 2018 [2] Matsuyama, 2018 [1]	Retrospective cohort	Japan (whole year)	537	ED patients aged ≥ 18 years whose body temperature was ≤ 35 °C	79 (66–87)	≤ 35 °C	Median 30.8 (IQR 28.2–32.6)	Whole cohort,* n* = 537: Acute medical illness: *n* = 271 (51%), Trauma: *n* = 73 (14%), Alcohol intoxication: *n* = 50 (9%), Drowning: *n* = 33 (6%), Self-harm: *n* = 34 (6%), Other: *n* = 137 (26%)
Sequeira, 2020 [12]	Retrospective cohort	USA (whole year)	203	ED patients aged > 18 years with suspected hypothermia. Patients not receiving rewarming treatment were excluded	Pre-policy group: mean 72 (SD 17.6), post-policy group: mean 66.7 (SD 17.9) ¢	< 35 °C	Pre-policy group: mean 33.7 (SD 1.4), post-policy group: mean 33.3 (SD 2.2)^b^	Whole cohort,* n *= 203: Altered mental status: *n* = 53 (26%), Hypoglycemia: *n* = 23 (11%), Infection: *n* = 41 (20%), Alcohol: *n* = 52 (26%), Other: *n* = 12 (6%), Congestive heart failure: *n* = 5 (2%), Hypothyroidism: *n* = 8 (4%), Pulmonary (non-infectious): *n* = 4 (2%), Gastrointestinal bleed: *n* = 4 (2%), Myocardial infarction: *n* = 1 (0.5%)
Takauji, 2021 [13]	Prospective observational cohort	Japan (winter)	1194	ED patients aged > 18 years whose body temperature was ≤ 35 °C	79 (68–87)	≤ 35 °C measured from the rectum, bladder or esophagus if available, otherwise from axilla and ears	30.8 (28.4–33.6)	Whole cohort,* n* = 1194: Acute medical illness: *n* = 595 (49%), which included:^a^ ◦ Infection (n = 116), ◦ Cerebrovascular disease (*n* = 72), ◦ Hypoglycemia (*n* = 65), ◦ Gastrointestinal disease (*n* = 61), ◦ Malnutrition (*n* = 52), ◦ Cardiac failure (*n* = 45), ◦ Hyperglycemia (*n* = 44), ◦ Ischemic cardiac disease (*n* = 36), ◦ Renal disease (*n* = 28), ◦ Endocrine disease (*n* = 14), ◦ Epilepsy (*n* = 5), ◦ Arrhythmia (*n* = 5), ◦ Others (*n* = 151) Trauma/submersion/distress: *n* = 164 (14%), Alcohol intoxication: *n* = 57 (5%), Drug: *n* = 25 (2%), Unknown: *n* = 270 (23%)

*IQR* Interquartile Range, *ED* emergency department, *UTI* urinary tract infection, *SSTI*  Skin and soft tissue infection

^a^The sum of the underlying acute medical illnesses overrides *n* = 595. Takauji et al. do not address this discrepancy. We interpreted this result that multiple subcauses within one patient were possible

^b^A guideline-based rewarming policy was implemented during the study period

Only the study of Morita et al. [2] and Matsuyama et al. [1] reported data solely regarding ED patients aged ≥ 65 years (Appendix 3, table A1). Internal disease was the most frequent cause of hypothermia in patients aged ≥ 65 years (46% up to 57%), and these incidences increased significantly with age (*p* value = 0.011) [2]. Internal diseases included stroke, seizure, Parkinson’s disease, thyroid disease, hypoglycemia, infectious disease, acute pancreatitis, uremia, malignant disease, bowel ischemia, and rhabdomyolysis [2]. The second most reported cause of hypothermia was the category ‘other’, which included iatrogenic, mountain accident, burn and malnutrition/infirmity causes [1, 2]. Incidences of alcohol intoxication, drowning and self-harm as underlying cause of hypothermia significantly decreased with age (all *p *values < 0.001). Furthermore, they also reported the incidences of underlying causes of hypothermia based on severity of hypothermia (Appendix 3, table A2). Severity of hypothermia did not significantly affect the incidences of underlying causes of hypothermia, except for the cause ‘drowning’, which incidence was significantly higher in mild (32–35 degrees Celsius) hypothermic patients compared to patients with a lower body temperature (*p* value = 0.001) [1].

Although we planned to perform a meta-analysis, this was not possible due to the heterogeneity in reported underlying causes.

## Case reports

Thirteen case reports and 22 conference abstracts were included in this review (Appendix 4, table A3). All included conference abstracts described a single patient. The age of patients ranged from 65 to 94 years. 9 out of 35 patients had a past medical history of memory loss or dementia, 2 of Parkinson’s disease, and 7 of a psychiatric disorder. Furthermore, seven patients had a history of chronic kidney disease, six of diabetes mellitus, and five of thyroid disease. The lowest body temperature reported was 23.1 degrees Celsius. Notable, 7 out of 9 patients known with memory loss or dementia and had a body temperature ≤ 32.0 degrees Celsius.

The most frequent reported underlying causes of hypothermia were thyroid failure (11 patients) and drug-induced hypothermia (11 patients). Drug-induced hypothermia was caused by the use of: quetiapine (2 patients), olanzapine (2), mirtazapine (1), lithium (1), amiodarone (1), metformin (1), secobarbital and pentobarbital (1), and alcohol (2).

## Discussion

This systematic review provided an overview of the existing literature on the incidences of underlying causes of hypothermia in older patients at the ED. We found that limited studies have been conducted regarding this topic, and all but one study were found to be of poor to medium quality. The few studies that have been published vary greatly in their reported underlying causes and its incidences. Common causes include acute medical illness, which includes infectious, endocrine, neurological, cardiac and gastro-intestinal diseases, trauma, alcohol intoxication and primary hypothermia. Less common causes include thyroid failure and drug-induced hypothermia. The study of Morita et al. [2] and Matsuyama et al. [1] demonstrated that the incidence of internal diseases as underlying cause of hypothermia increases with age and the incidence of external factors like alcohol intoxication, drowning and self-harm decreases with age. However, solely the study of Morita et al. [2] and Matsuyama et al. [1] has reported these associations thus far. Overall, we graded the overall quality of evidence of the included studies in this review based on the GRADE framework as low.

There was a wide variety in the incidences of underlying causes of hypothermia between studies. This might be explained due to the fact that the studies were performed in different countries with different climates, living circumstances, health care systems and infrastructures, which may affect the incidence of a specific disease. For example, as primary hypothermia is often associated with exposure to cold environments, its incidence is likely to be higher in countries with a polar climate compared to a tropical climate [16]. However, hypothermia can also occur in warmer climates, especially in winters, suggesting older adults are not only prone to absolute, but also to relatively low ambient temperatures [17, 18]. How absolute and relative low ambient temperatures are mutually related needs yet to be established.

Remarkably, only the study of Cassar et al. [14] explicitly reported data regarding primary hypothermia. However, this study was performed in Malta, which has a Mediterranean climate, thus making a relatively high prevalence of primary hypothermia compared to other studies due to extreme weather conditions less explicable. An alternative feasible explanation is that the exact classification of primary and secondary hypothermia varied per study protocol. A recent Danish study showed that 57% of hypothermic hospitalized patients had at least two diagnoses which contributed to their hypothermic state [3].The study protocols that describe how to determine which diagnosis is the primary cause of hypothermia most likely varied between studies. For example, a frail older patient with a mild trauma who had been lying on the ground for days may have been classified as primary hypothermia in one study, but as secondary hypothermia due to trauma in another study.

Another noteworthy observation in our results was that the median body temperatures of patients in the studies of Morita et al. [2], Matsuyama et al. [1], and Takauji et al. [13] were relatively low with a median of both 30.8 degrees Celsius. We expected the distribution of body temperature in hypothermic ED patients to be skewed, meaning that many patients will suffer from mild hypothermia, but when the severity of hypothermia increases, the frequency of affected patients decreases. A plausible explanation for this relatively low median body temperature might be that the study population of Morita et al. [2] and Matsuyama et al. [1] was selected based on patients who had an International Classification of Diseases, 10th Revision (ICD-10) code T68 (hypothermia) registered in their medical record. This may have resulted in selection bias, as treating physicians may not be inclined to register this ICD-10 code to patients whose main symptom was not hypothermia or if hypothermia was relatively mild. However, the study of Takauji et al. [13] did not report the use of ICD-10 codes, which makes their relatively low median body temperature a remarkable finding.

To our knowledge, this is the first systematic review regarding the underlying causes of hypothermia in older ED patients, with a thorough approach including case reports and conference abstracts. However, our result should be seen in the light of some limitations. Our search was limited by language restrictions, since we only included articles in English, German or Dutch. However, in our opinion, the main language in science, English, was included. Furthermore, a meta-analysis would have been of additional value; but the heterogeneity between studies was too large to reliable pool results.

In conclusion, hypothermia is a serious condition in older adults with high rates of morbidity and mortality, especially in frail older patients [2, 4]. Prompt recognition and treatment of the underlying cause are needed in order to minimize adverse events. A wide variety of factors can cause hypothermia, and its incidences vary per region. Common causes that should not be missed include infectious, endocrine, neurological, cardiac and gastro-intestinal diseases, trauma, alcohol intoxication and primary hypothermia. More rare causes include thyroid failure and drug-induced hypothermia; however, these may not be missed as these can lead to severe illness. There is some evidence that suggests that incidences of internal disease as underlying cause of hypothermia increase with age and incidences of causes like alcohol intoxication, drowning and self-harm decrease with age. If an older patient presents itself to the ED with hypothermia, a thorough diagnostic workup is needed in order to identify and treat the underlying cause rapidly. This systematic review may assist physicians in making treatment decisions for older patients presenting to the ED with hypothermia. However, more research is needed in order to further determine the incidences of underlying causes of hypothermia. Future research should focus specifically on older patients and should include patients from different regions in order to determine how factors such as climate, living circumstances, health care system, and infrastructure may affect the incidences of underlying causes of hypothermia.

## Supplementary Information

Below is the link to the electronic supplementary material.

Supplementary file1 (DOCX 59 KB)

## Data Availability

Additional data that supports the findings of this research are available from the authors upon request.
