# Supplementary material for: Incidences of underlying causes of hypothermia in older patients in the emergency department: a systematic review
Source: Eur Geriatr Med. 2023 May 16;14(3):411–20. doi: 10.1007/s41999-023-00791-0 (PMC10261225; doi:10.1007/s41999-023-00791-0)
Supplement: Supplementary file 1 — Supplementary file1 (DOCX 59 KB) [file 41999_2023_791_MOESM1_ESM.docx]

**Incidences of underlying causes of hypothermia in older patients in the emergency department: a systematic review**

**Appendix**

**Appendix 1** – PRISMA checklists

**Appendix 2** - Complete search strategy per database

**Appendix 3** - Etiology of hypothermia (categorized by age or severity of hypothermia)

**Appendix 4** - Case reports of hypothermic older patients

**Appendix references**

**Appendix 1 – PRISMA checklists**

*PRISMA 2020 Checklist*

| **Section and Topic** | **Item #** | **Checklist item** | **Location where item is reported** |
| --- | --- | --- | --- |
| **TITLE** | | |  |
| Title | 1 | Identify the report as a systematic review. | Page 1 |
| **ABSTRACT** | | |  |
| Abstract | 2 | See the PRISMA 2020 for Abstracts checklist. | Included below |
| **INTRODUCTION** | | |  |
| Rationale | 3 | Describe the rationale for the review in the context of existing knowledge. | Page 3 |
| Objectives | 4 | Provide an explicit statement of the objective(s) or question(s) the review addresses. | Page 4 |
| **METHODS** | | |  |
| Eligibility criteria | 5 | Specify the inclusion and exclusion criteria for the review and how studies were grouped for the syntheses. | Page 4-5 |
| Information sources | 6 | Specify all databases, registers, websites, organisations, reference lists and other sources searched or consulted to identify studies. Specify the date when each source was last searched or consulted. | Page 4-5 |
| Search strategy | 7 | Present the full search strategies for all databases, registers and websites, including any filters and limits used. | Page 4-5  Supplement 2 |
| Selection process | 8 | Specify the methods used to decide whether a study met the inclusion criteria of the review, including how many reviewers screened each record and each report retrieved, whether they worked independently, and if applicable, details of automation tools used in the process. | Page 4-5 |
| Data collection process | 9 | Specify the methods used to collect data from reports, including how many reviewers collected data from each report, whether they worked independently, any processes for obtaining or confirming data from study investigators, and if applicable, details of automation tools used in the process. | Page 6 |
| Data items | 10a | List and define all outcomes for which data were sought. Specify whether all results that were compatible with each outcome domain in each study were sought (e.g. for all measures, time points, analyses), and if not, the methods used to decide which results to collect. | Page 6 |
|  | 10b | List and define all other variables for which data were sought (e.g. participant and intervention characteristics, funding sources). Describe any assumptions made about any missing or unclear information. | Page 6 |
| Study risk of bias assessment | 11 | Specify the methods used to assess risk of bias in the included studies, including details of the tool(s) used, how many reviewers assessed each study and whether they worked independently, and if applicable, details of automation tools used in the process. | Page 6 |
| Effect measures | 12 | Specify for each outcome the effect measure(s) (e.g. risk ratio, mean difference) used in the synthesis or presentation of results. | N/A |
| Synthesis methods | 13a | Describe the processes used to decide which studies were eligible for each synthesis (e.g. tabulating the study intervention characteristics and comparing against the planned groups for each synthesis (item #5)). | Page 4-6 |
|  | 13b | Describe any methods required to prepare the data for presentation or synthesis, such as handling of missing summary statistics, or data conversions. | N/A |
|  | 13c | Describe any methods used to tabulate or visually display results of individual studies and syntheses. | Page 6 |
|  | 13d | Describe any methods used to synthesize results and provide a rationale for the choice(s). If meta-analysis was performed, describe the model(s), method(s) to identify the presence and extent of statistical heterogeneity, and software package(s) used. | Page 6-7 |
|  | 13e | Describe any methods used to explore possible causes of heterogeneity among study results (e.g. subgroup analysis, meta-regression). | N/A |
|  | 13f | Describe any sensitivity analyses conducted to assess robustness of the synthesized results. | N/A |
| Reporting bias assessment | 14 | Describe any methods used to assess risk of bias due to missing results in a synthesis (arising from reporting biases). | N/A |
| Certainty assessment | 15 | Describe any methods used to assess certainty (or confidence) in the body of evidence for an outcome. | Page 6 |
| **RESULTS** | | |  |
| Study selection | 16a | Describe the results of the search and selection process, from the number of records identified in the search to the number of studies included in the review, ideally using a flow diagram. | Page 7-8  Figure 1 |
|  | 16b | Cite studies that might appear to meet the inclusion criteria, but which were excluded, and explain why they were excluded. | Figure 1 |
| Study characteristics | 17 | Cite each included study and present its characteristics. | Page 9-12  Table 1  Supplement 4 |
| Risk of bias in studies | 18 | Present assessments of risk of bias for each included study. | Page 6  Figure 2 |
| Results of individual studies | 19 | For all outcomes, present, for each study: (a) summary statistics for each group (where appropriate) and (b) an effect estimate and its precision (e.g. confidence/credible interval), ideally using structured tables or plots. | Table 1 |
| Results of syntheses | 20a | For each synthesis, briefly summarise the characteristics and risk of bias among contributing studies. | N/A |
|  | 20b | Present results of all statistical syntheses conducted. If meta-analysis was done, present for each the summary estimate and its precision (e.g. confidence/credible interval) and measures of statistical heterogeneity. If comparing groups, describe the direction of the effect. | Table 1 |
|  | 20c | Present results of all investigations of possible causes of heterogeneity among study results. | N/A |
|  | 20d | Present results of all sensitivity analyses conducted to assess the robustness of the synthesized results. | N/A |
| Reporting biases | 21 | Present assessments of risk of bias due to missing results (arising from reporting biases) for each synthesis assessed. | N/A |

| Certainty of evidence | 22 | Present assessments of certainty (or confidence) in the body of evidence for each outcome assessed. | Page 14 |
| --- | --- | --- | --- |
| **DISCUSSION** | | |  |
| Discussion | 23a | Provide a general interpretation of the results in the context of other evidence. | Page 14-15 |
|  | 23b | Discuss any limitations of the evidence included in the review. | Page 14-16 |
|  | 23c | Discuss any limitations of the review processes used. | Page 16 |
|  | 23d | Discuss implications of the results for practice, policy, and future research. | Page 16-17 |
| **OTHER INFORMATION** | | |  |
| Registration and protocol | 24a | Provide registration information for the review, including register name and registration number, or state that the review was not registered. | Page 4 |
|  | 24b | Indicate where the review protocol can be accessed, or state that a protocol was not prepared. | Page 4 |
|  | 24c | Describe and explain any amendments to information provided at registration or in the protocol. | N/A |
| Support | 25 | Describe sources of financial or non-financial support for the review, and the role of the funders or sponsors in the review. | Page 18 |
| Competing interests | 26 | Declare any competing interests of review authors. | Page 18 |
| Availability of data, code and other materials | 27 | Report which of the following are publicly available and where they can be found: template data collection forms; data extracted from included studies; data used for all analyses; analytic code; any other materials used in the review. | N/A |

*From:*  Page MJ, McKenzie JE, Bossuyt PM, Boutron I, Hoffmann TC, Mulrow CD, et al. The PRISMA 2020 statement: an updated guideline for reporting systematic reviews. BMJ 2021;372:n71. doi: 10.1136/bmj.n71

For more information, visit: <http://www.prisma-statement.org/>

*PRISMA 2020 for Abstracts Checklist*

| **Section and Topic** | **Item #** | **Checklist item** | **Reported (Yes/No)** |
| --- | --- | --- | --- |
| **TITLE** | | |  |
| Title | 1 | Identify the report as a systematic review. | Yes |
| **BACKGROUND** | | |  |
| Objectives | 2 | Provide an explicit statement of the main objective(s) or question(s) the review addresses. | Yes |
| **METHODS** | | |  |
| Eligibility criteria | 3 | Specify the inclusion and exclusion criteria for the review. | Yes |
| Information sources | 4 | Specify the information sources (e.g. databases, registers) used to identify studies and the date when each was last searched. | Yes |
| Risk of bias | 5 | Specify the methods used to assess risk of bias in the included studies. | Yes |
| Synthesis of results | 6 | Specify the methods used to present and synthesise results. | Yes |
| **RESULTS** | | |  |
| Included studies | 7 | Give the total number of included studies and participants and summarise relevant characteristics of studies. | Yes |
| Synthesis of results | 8 | Present results for main outcomes, preferably indicating the number of included studies and participants for each. If meta-analysis was done, report the summary estimate and confidence/credible interval. If comparing groups, indicate the direction of the effect (i.e. which group is favoured). | Yes |
| **DISCUSSION** | | |  |
| Limitations of evidence | 9 | Provide a brief summary of the limitations of the evidence included in the review (e.g. study risk of bias, inconsistency and imprecision). | Yes |
| Interpretation | 10 | Provide a general interpretation of the results and important implications. | Yes |
| **OTHER** | | |  |
| Funding | 11 | Specify the primary source of funding for the review. | Yes |
| Registration | 12 | Provide the register name and registration number. | In Methods |

*From:*  Page MJ, McKenzie JE, Bossuyt PM, Boutron I, Hoffmann TC, Mulrow CD, et al. The PRISMA 2020 statement: an updated guideline for reporting systematic reviews. BMJ 2021;372:n71. doi: 10.1136/bmj.n71

For more information, visit: <http://www.prisma-statement.org/>

**Appendix 2 - Complete search strategy per database**

*MEDLINE*

(Elderly [Title/Abstract] OR community-dwelling[Title/Abstract] OR geriatric [Title/Abstract] OR “mini-mental state”[Title/Abstract] OR alzheimer [Title/Abstract] OR alzheimer’s[Title/Abstract] OR alzheimers[Title/Abstract] OR mmse[Title/Abstract] OR caregivers[Title/Abstract]OR falls [Title/Abstract] OR Adl[Title/Abstract] OR Frailty[ Title/Abstract] OR Gds[Title/Abstract] OR Ageing[Title/Abstract] OR “hip fractures “[Title/Abstract] OR elders[Title/Abstract] OR Frail[Title/Abstract] OR Mci[Title/Abstract] OR Demented[Title/Abstract] OR Psychogeriatrics[Title/Abstract] OR “cognitive impairment”[Title/Abstract] OR “postmenopausal women”[Title/Abstract] OR comorbidities[Title/Abstract] OR dementia[Title/Abstract] OR aging[Title/Abstract] OR older [Title/Abstract] OR “daily living”[Title/Abstract] OR “cognitive decline”[Title/Abstract] OR “cognitive impairment”[Title/Abstract] OR resident[Title/Abstract] OR “cognitive functioning”[Title/Abstract] OR “old people”[Title/Abstract] OR nursing homes OR “Geriatric assessment” OR aging OR “frail elderly” OR “Alzheimer disease” OR “homes for the aged” OR “cognition disorders” OR dementia OR “Activities of daily living” OR “aged, 80 and over ”)

AND

(hypotherm*[Title/Abstract] OR “low body temperature” [Title/Abstract] OR “Hypothermia”)

 AND

(Emergency[Title/Abstract] OR ED[Title/Abstract] OR ER[Title/Abstract] OR “Critical Care”[Title/Abstract] OR acute[Title/Abstract] OR accident*[Title/Abstract] OR trauma*[Title/Abstract] OR “Emergency Service, Hospital”)

*The Cochrane Library*

#1 (Elderly):ti,ab,kw OR (community-dwelling):ti,ab,kw OR (geriatric):ti,ab,kw OR (mini-mental state):ti,ab,kw OR (alzheimer):ti,ab,kw OR (alzheimer’s):ti,ab,kw OR (alzheimers):ti,ab,kw OR (mmse):ti,ab,kw OR (caregivers):ti,ab,kw OR (falls):ti,ab,kw OR (Adl):ti,ab,kw OR (Frailty):ti,ab,kw OR (Gds):ti,ab,kw OR (Ageing):ti,ab,kw OR (hip fractures):ti,ab,kw OR (elders):ti,ab,kw OR (Frail):ti,ab,kw OR (Mci):ti,ab,kw OR (Demented):ti,ab,kw OR (Psychogeriatrics):ti,ab,kw OR (cognitive impairment):ti,ab,kw OR (postmenopausal women):ti,ab,kw OR (comorbidities):ti,ab,kw OR (dementia):ti,ab,kw OR (aging):ti,ab,kw OR (older):ti,ab,kw OR (daily living):ti,ab,kw OR (cognitive decline):ti,ab,kw OR (cognitive impairment):ti,ab,kw OR (resident):ti,ab,kw OR (cognitive functioning):ti,ab,kw OR (old people):ti,ab,kw

#2 MeSH descriptor: [Nursing Homes] explode all trees

#3 MeSH descriptor: [Geriatric assessment] explode all trees

#4 MeSH descriptor: [Aging] explode all trees

#5 MeSH descriptor: [Frail Elderly] explode all trees

#6 MeSH descriptor: [Alzheimer Disease] explode all trees

#7 MeSH descriptor: [Homes for the Aged] explode all trees

#8 MeSH descriptor: [Cognition Disorders] explode all trees

#9 MeSH descriptor: [Dementia] explode all trees

#10 MeSH descriptor: [Activities of Daily Living] explode all trees

#11 MeSH descriptor: [Aged, 80 and over] explode all trees

#12 #1 OR #2 OR #3 OR #4 OR #5 OR #6 OR #7 OR #8 OR #9 OR #10 OR #11

#13 (hypotherm*):ti,ab,kw OR (low body temperature):ti,ab,kw

#14 MeSH descriptor: [Hypothermia] explode all trees

#15 #13 OR #14

#16 (Emergency):ti,ab,kw OR (ED):ti,ab,kw OR (ER):ti,ab,kw OR (Critical Care):ti,ab,kw OR (acute):ti,ab,kw OR (accident*):ti,ab,kw OR (trauma*):ti,ab,kw

#17 MeSH descriptor: [Emergency Service, Hospital] explode all trees

#18 #16 OR #17

#19 #12 AND #15 AND #18

*Embase*

(Elderly:ti,ab,kw OR community-dwelling:ti,ab,kw OR geriatric:ti,ab,kw OR ‘mini-mental state’:ti,ab,kw OR Alzheimer*:ti,ab,kw OR mmse:ti,ab,kw OR caregivers:ti,ab,kw OR falls:ti,ab,kw OR Adl:ti,ab,kw OR Frailty:ti,ab,kw OR Gds:ti,ab,kw OR Ageing:ti,ab,kw OR ‘hip fractures’:ti,ab,kw OR elders:ti,ab,kw OR Frail:ti,ab,kw OR Mci:ti,ab,kw OR Demented:ti,ab,kw OR Psychogeriatrics:ti,ab,kw OR ‘cognitive impairment’:ti,ab,kw OR ‘postmenopausal women’:ti,ab,kw OR comorbidities:ti,ab,kw OR dementia:ti,ab,kw OR aging:ti,ab,kw OR older:ti,ab,kw OR ‘daily living’:ti,ab,kw OR ‘cognitive decline’:ti,ab,kw OR ‘cognitive impairment’:ti,ab,kw OR resident:ti,ab,kw OR ‘cognitive functioning’:ti,ab,kw OR ‘old people‘:ti,ab,kw OR ‘nursing home’/exp OR 'geriatric assessment'/exp OR aging/exp OR ‘frail elderly’/exp OR 'Alzheimer disease'/exp OR 'home for the aged'/exp OR 'cognitive defect'/exp OR dementia/exp OR 'daily life activity'/exp OR 'very elderly'/exp)

AND

(hypotherm*:ti,ab,kw OR ‘low body temperature’:ti,ab,kw OR hypothermia/exp)

 AND

(Emergency:ti,ab,kw OR ED:ti,ab,kw OR ER:ti,ab,kw OR ‘Critical Care’:ti,ab,kw OR acute:ti,ab,kw OR accident*:ti,ab,kw OR trauma*:ti,ab,kw OR ‘emergency ward’/exp)

**Appendix 3 – Etiology of hypothermia (categorized by age or severity of hypothermia)**

**Table A1.** Etiology of hypothermia, categorized per age group^∆^

| **Associated condition** | **Adult (aged 18-64 years)**  **n = 113** | **Young-old (aged 65-79 years)**  **n = 164** | **Old-old (aged ≥ 80 years)**  **n = 260** | **P values** |
| --- | --- | --- | --- | --- |
| Internal diseases | 47 (42%) | 76 (46%) | 148 (57%) | 0.011* |
| Trauma | 10 (9%) | 26 (16%) | 37 (14%) | 0.227 |
| Alcohol intoxication | 19 (17%) | 23 (14%) | 8 (3%) | <0.001* |
| Drowning including immersion | 19 (17%) | 8 (5%) | 6 (2%) | <0.001* |
| Self-harm | 20 (18%) | 10 (6%) | 4 (2%) | <0.001* |
| Other | 26 (23%) | 30 (18%) | 81 (31%) | 0.010* |

*∆ Table adapted from Morita et al.[1] and Matsuyama et al.[2], * = significant p-value.*

**Table A2.** Etiology of hypothermia, categorized by severity of hypothermia^∆^

| **Associated condition** | **Mild hypothermia (32-35°C)**  **n = 192** | **Moderate hypothermia (28-31°C)**  **n = 227** | **Severe hypothermia (<28°C)**  **n = 118** | **P values** |
| --- | --- | --- | --- | --- |
| Acute medical illness | 90 (47%) | 125 (55%) | 57 (48%) | 0.249 |
| Trauma | 27 (14%) | 31 (14%) | 15 (13%) | 0.944 |
| Alcohol intoxication | 23 (12%) | 17 (8%) | 10 (9%) | 0.271 |
| Drowning | 20 (10%) | 4 (2%) | 9 (8%) | 0.001* |
| Self-harm | 18 (9%) | 11 (5%) | 5 (4%) | 0.095 |
| Other | 47 (25%) | 66 (29%) | 24 (20%) | 0.193 |

*∆ Table adapted from Morita et al.[1] and Matsuyama et al.[2], * = significant p-value.*

**Appendix 4 – Case reports of hypothermic older patients**

**Table A3.** Case reports and conference abstracts of etiology of hypothermia in older patients at the emergency department

| **First author, year** | **Study design** | **Patient characteristics** | **Age in years** | **Body temperature in °C at ED arrival (method of measurement if mentioned)** | **Underlying cause** |
| --- | --- | --- | --- | --- | --- |
| Woehrle, 2018 [3] | Case report | Woman with history of cachexia and dementia | 83 | 23.1 (rectal) | Hypothyroidism |
| Ngwainmbi, 2020 [4] | Conference abstract | Man with unknown medical history who was found lying outdoors while temperatures were below freezing | 65 | 27.0 | Myxedema coma and accidental hypothermia |
| Charoenpong, 2013 [5] | Conference abstract | Woman with worsened memory loss found unresponsive on the street | 74 | 28.1 (rectal) | Hypothyroidism |
| Schattner, 2021 [6] | Case report | Woman with history of Alzheimer’s dementia, falls, hypothyroidism (on replacement), HT, and chronic AF | 89 | 28.5 (rectal) | Quetiapine-induced |
| Shekar, 2015 [7] | Conference abstract | Woman with history of HT, CHF, CKD, type 2 DM not on insulin, CVA, and dementia | 94 | 28.8 | Hypermagnesemia and hypoglycemia |
| Sim, 2000 [8] | Case report | Man found lying partially immersed in padi field in subtropics | 86 | 29.3 (tympanic) | Accidental hypothermia and aspiration pneumonia |
| Yamashita, 2012 [9] | Case report | Man with history of partial tetraplegia, depression, and deep vein thrombosis | 75 | 29.5 (rectal) | Accidental hypothermia |
| Sehara, 2009 [10] | Case report | Woman with Parkinson’s disease | 80 | 29.7 | Accidental hypothermia, possible autonomic dysfunction |
| Kogan, 2011 [11] | Case report | Woman with unknown medical history | 84 | 30.0 (rectal) | Myxedema coma |
| Sforza, 2019 [12] | Case report | Man with history of dementia and HT | 83 | 30.1 (rectal) | Pneumonia |
| Neves, 2017 [13] | Conference abstract | Man with history of CVA, dyslipidemia, and CAD | 88 | 30.5 | Myxedema coma |
| Monti, 2018 [14] | Case report | Italian woman with history of generalized anxiety disorder using olanzapine for two years | 83 | 30.9 (rectal) | Olanzapine |
| Ueda, 2019 [15] | Case report | Japanese man with history of Hashimoto's thyroiditis | 84 | 31.0 (rectal) | Myxedema coma |
| Regula, 2020 [16] | Conference abstract | Black woman with history of schizophrenia and dementia | 69 | 31.1 | Quetiapine induced hypoglycemia and hypothermia |
| Kansagra, 2013 [17] | Case report and literature review | Man with history of insulin-dependent DM, CKD stage III, and bipolar disease | 80 | 31.2 (rectal) | Olanzapine use in the setting of chronic kidney disease complicated by acute kidney injury |
| Hiltbold, 2015 [18] | Conference abstract | Woman with history of DM, HT, and paroxysmal AF | 85 | 32.0 | Myxedema coma |
| Price, 2019 [19] | Conference abstract | Man with history of dementia living in a local skilled nursing facility | 66 | 32.0 | Spontaneous periodic hypothermia |
| Qamar, 2020 [20] | Conference abstract | Man with history of dementia and alcohol use | 79 | 32.3 | Acute esophageal necrosis |
| Ali, 2019 [21] | Conference abstract | Ethiopian woman with history of hypothyroidism and chronic lymphedema, noncompliant with levothyroxine | 66 | 32.5 | Myxedema coma |
| Kuroski, 2019 [22] | Conference abstract | Woman with history of CHF, hypertension, paroxysmal AF, and hypothyroidism | 90 | 32.5 | Myxedema coma, precipitated by amiodarone |
| Orsi, 2010 [23] | Conference abstract | Woman with DM and CKD stage III | 75 | 33.6 (rectal) | Acute pancreatitis and metformin toxicity |
| Flood, 2017 [24] | Conference abstract | Man with unknown medical history | 82 | 33.9 | Diogenes syndrome and pneumonia |
| Bava, 2021 [25] | Conference abstract | Italian woman with history of HT, previous breast cancer, and cervical cancer | 86 | 34.0 | Takotsubo cardiomyopathy due to fear of COVID |
| Thom, 2011 [26] | Conference abstract | Man from UK with possible depression and history of ischemic heart disease, AF, Barrett’s esophagus, CKD and BPH | 84 | 35.4 | Absence seizures secondary to mirtazapine |
| Shah, 2018 [27] | Conference abstract | Woman with history of hyperthyroidism using iodine supplements | 73 | 35.0 | Hypothyroidism secondary to excessive iodine intake |
| Wilson, 2007 [28] | Case report | Man from UK with history of longstanding depression and deliberate drug overdoses | 80 | 35.3 (tympanic) | Acute alcohol intoxication |
| Nat, 2013 [29] | Conference abstract | Man with Parkinson’s disease, DM, and bipolar disorder | 72 | 35.4 | Chronic lithium toxicity |
| Kulkarni, 2019 [30] | Conference abstract | Man with history of HT and alcohol abuse | 70 | 35.5 | Acute alcohol withdrawal-induced takotsubo cardiomyopathy |
| Minami, 2016 [31] | Conference abstract | Japanese woman with history of acute pancreatitis, CKD, anemia of chronic disease, RA, liver cirrhosis, HT, and gastric cancer | 78 | 35.6 | Immune mediated diabetes mellitus type 1 |
| Katz, 2021 [32] | Case report | Women with medical history of Non-Hodgkin's splenic marginal zone lymphoma, hemolytic anemia, hepatocellular carcinoma, HT, neurogenic bladder, osteoarthritis, and shingles | 94 | 35.7 (temporal) | Secobarbital and pentobarbital intoxication |
| Harrison, 2014 [33] | Conference abstract | Man with history of advanced dementia, CAD, and HT | 87 | 35.7 | Myxedema coma |
| Shah, 2016 [34] | Case report | Man with history of HT, CVA, and glaucoma | 79 | No detectable temperature (rectal) | Sepsis with S. aureus |
| Sharma, 2018 [35] | Conference abstract | Woman with history of ESRD on HD via AV fistula | 82 | Hypothermic | Native aortic valve endocarditis causing septic shock due to S. epidermidis |
| Deutch, 2020 [36] | Conference abstract | Women with history of alcoholic cirrhosis, CKD, DM, and squamous cell esophageal cancer | 68 | Hypothermic | Septic shock due to epidural abscess from erosion of esophageal stent |
| Tsang, 2021 [37] | Conference abstract | Man who represented to the ED three days after admission for pneumonia, during which a sodium phosphate enema was given for constipation | 83 | Hypothermic | Rectal perforation after enema |

*Abbreviations: ED = emergency department, HT = hypertension, AF = atrial fibrillation, CHF = congestive heart failure, CKD = chronic kidney disease, DM = diabetes mellitus, CVA = cerebrovascular accident, CAD = coronary artery disease, BPH = benign prostatic hypertrophy, ESRD = end stage renal disease, HD = hemodialysis, AV = arteriovenous.*

**Appendix references**

1. Morita S, Matsuyama T, Ehara N, et al. Prevalence and outcomes of accidental hypothermia among elderly patients in Japan: Data from the J-Point registry. Geriatr Gerontol Int. 2018;18(10):1427-32.

2. Matsuyama T, Morita S, Ehara N, et al. Characteristics and outcomes of accidental hypothermia in Japan: the J-Point registry. Emerg Med J. 2018;35(11):659-66.

3. Woehrle T, Lichtenauer U, Bayer A, et al. Misleading symptoms and successful noninvasive rewarming of a patient with severe hypothermia (23.1 degrees C). Anaesthesist. 2018;67(12):931-5.

4. Ngwainmbi D, Durai P, Lopez LR, et al. HYPOTHERMIC EMERGENCY. Chest. 2020;158(4).

5. Charoenpong P, Clarke C, Shahwan K, et al. Complication of active rewarming in hypothermia from hypothyroidism. Crit Care Med. 2013;41(12).

6. Schattner A, Dubin I. Hypothermia induced by quetiapine. Postgrad Med J. 2021;97(1145):140.

7. Shekar C, Puri S. Hypermagnesemia can stop the heart. J Gen Intern Med. 2015;30.

8. Sim MM, Kuo YC. Accidental hypothermia in the subtropics. Am J Emerg Med. 2000;18(3):357-8.

9. Yamashita K, Suganuma K, Funase Y, et al. Elevation of thyrotropin upon accidental hypothermia in an elderly man. Thyroid. 2012;22(12):1291-3.

10. Sehara Y. Hypothermia with Osborn waves in Parkinson's disease. Intern Med. 2009;48(8):615-8.

11. Kogan A, Kassif Y, Shadel M, et al. Severe hypothermia in myxoedema coma: a rewarming by extracorporeal circulation. Emerg Med Australas. 2011;23(6):773-5.

12. Sforza A, Carlino MV, Mancusi C, et al. Bradycardia in elderly patients: Look at the body temperature. Ital J Med. 2019;13(4):241-3.

13. Neves JS, Oliveira AI, Varela A, et al. Myxedema coma secondary to hypopituitarism in a patient without previously known pituitary dysfunction. Endocr Rev. 2017;38(3).

14. Monti M, Mommi V, Forte MB, et al. Olanzapine-associated hypothermia: A case report of a rare event. Ital J Med. 2018;12(1):67-9.

15. Ueda K, Kiyota A, Tsuchida M, et al. Successful treatment of myxedema coma with a combination of levothyroxine and liothyronine. Endocr J. 2019;66(5):469-74.

16. Vadhar PRMGB, Halanych J. QUETIAPINE-INDUCED HYPOGLYCEMIA AND HYPOTHERMIA MASQUERADING AS SEPSIS. Chest. 2020;158(4).

17. Kansagra A, Patel S, Wilcox SR. Prolonged hypothermia due to olanzapine in the setting of renal failure: A case report and review of the literature. Ther Adv Psychopharmacol. 2013;3(6):335-9.

18. Hiltbold AE, Mohiuddin A. The coma you can't mix up. J Gen Intern Med. 2015;30

19. Price HN, Jacobs J, Hajduczok A, et al. A cool case report: Spontaneous periodic hypothermia. J G Intern Med. 2019;34(2).

20. Qamar K, Arora A, Gill I, et al. Black esophagus: An unusual cause of massive upper gi bleeding. Am J Gastroenterol. 2020;115.

21. Ali S, Shariff M, Maknojia A, et al. CARDIAC TAMPONADE: A RARE MANIFESTATION OF MYXEDEMA COMA. Chest. 2019;156(4).

22. Kuroski J, Kolenda M, Cooley B. Presentation of myxedema coma to the emergency department following recent initiation of amiodarone. Crit Care Med. 2019;47(1).

23. Orsi D, Dudaie R, Dicpinigaitis P. Acute pancreatitis associated with metformin toxicity. Crit Care Med. 2010;38(A271).

24. Flood Z, Lavelle M, Daly T. Recognising diogenes syndrome: A case report. Ir J Med Sci. 2017;186(6).

25. Bava A, Postorino S, Lanteri S, et al. A case of takotsubo cardiomyopathy due to fear of COVID. Eur Heart J. 2021;23.

26. Thom V, Solanki T. Hypothermia-an unusual iatrogenic cause. Eur Geriatr Med. 2011;2(S111).

27. Shah M, Sosa Pgan M. You can never have too much of a good thing, or can you? A case of severe hypothyroidism secondary to excessive iodine intake. Thyroid. 2018;A122.

28. Wilson E, Waring WS. Severe hypotension and hypothermia caused by acute ethanol toxicity. Emerg Med J. 2007;24(2):e7.

29. Nat AS, Nat AS, Rane M, et al. Caution:Lithium induced multiorgan dysfunction in the elderly. J Gen Intern Med. 2013;28(S305).

30. Kulkarni M, Kulkarni A, Healy J, et al. ACUTE ALCOHOL WITHDRAWAL-INDUCED TAKOTSUBO CARDIOMYOPATHY: A RARE PRESENTATION. Chest. 2019;156(4).

31. Minami R, Constantine S. Elderly onset of diabetes mellitus type 1. J Gen Intern Med. 2016;31(2).

32. Katz KD, Koons A, Makar G, et al. Old and Cold: A Novel Case of Combined Secobarbital and Pentobarbital Poisoning in an Elderly Woman. Cureus. 2021;13(1):e12446.

33. Harrison L, Rahmat L. The façade of familiarity-a missed case of myxedema coma. J Gen Intern Med. 2014;29.

34. Shah R, Rashid A, Mizeracki A. An elderly unresponsive patient. J Am Med Assoc. 2016;315(17):1896-7.

35. Sharma A, Vadehra D, Montesano P, et al. Staphylococcus epidermidis and hemodialysis: A deadly duo causing native valve endocarditis. Am J Respir Crit Care Med. 2018;197.

36. Deutch Z, Castro C, Krewson T, et al. EPIDURAL ABSCESS FROM EROSION OF ESOPHAGEAL STENT: A CRYPTIC CAUSE OF SEPTIC SHOCK. Chest. 2020;158(4).

37. Tsang C, Baker J, Cheong J, et al. Rectal perforation and necrosis associated with fleet enema. Dis Colon Rectum. 2021;64(5).
